# Correction: Evaluating the generalisability of region-naïve machine learning algorithms for the identification of epilepsy in low-resource settings

**DOI:** 10.1371/journal.pdig.0000841

**Published:** 2025-04-17

**Authors:** Ioana Duta, Symon M. Kariuki, Anthony K. Ngugi, Angelina Kakooza Mwesige, Honorati Masanja, Daniel M. Mwanga, Seth Owusu-Agyei, Ryan Wagner, J. Helen Cross, Josemir W. Sander, Charles R. Newton, Arjune Sen, Gabriel Davis Jones

There are errors in the authors’ affiliations. The correct affiliations are listed below:

Ioana Duta^1,2^, Symon M. Kariuki^3,4,5^, Anthony K. Ngugi^4,6^, Angelina Kakooza Mwesige^7^, Honorati Masanja^8^, Daniel M. Mwanga^9,10^, Seth Owusu-Agyei^11,12^, Ryan Wagner^13^, J. Helen Cross^14^, Josemir W. Sander^15,16,17^, Charles R. Newton^1,3,4,18^, Arjune Sen^1^, Gabriel Davis Jones^1,2,19^

**1** Oxford Epilepsy Research Group, Nuffield Department of Clinical Neurosciences, John Radcliffe Hospital, Oxford, United Kingdom, **2** Oxford Digital Health Labs, Nuffield Department of Women’s and Reproductive Health, The University of Oxford, John Radcliffe Hospital, Oxford, United Kingdom, **3** KEMRI/Wellcome Trust Research Programme, Centre for Geographic Medicine Research – Coast, Kilifi, Kenya, **4** Studies of Epidemiology of Epilepsy in Demographic Surveillance Systems (SEEDS) – INDEPTH Network, Accra, Ghana, **5** Department of Public Health, Pwani University, Kilifi, Kenya, **6** Department of Population Health, Aga Khan University, Nairobi, Kenya, **7** Department of Paediatrics and Child Health, Makerere University College of Health Sciences, Kampala, Uganda, **8** Ifakara Health Institute, Ifakara, Tanzania, **9** Data Synergy and Evaluations, African Population and Health Research Center, Nairobi, Kenya, **10** Department of Mathematics, University of Nairobi, Nairobi, Kenya, **11** Kintampo Health Research Centre, Kintampo, Ghana, **12** Institute of Health Research, University of Health and Allied Sciences, Ho, Ghana, **13** MRC/Wits Rural Public Health & Health Transitions Research Unit (Agincourt), School of Public Health, Faculty of Health Sciences, University of the Witwatersrand, Johannesburg, South Africa, **14** Developmental Neurosciences, University College London, UCL- Great Ormond Street Institute of Child Health, London, United Kingdom, **15** Department of Clinical & Experimental Epilepsy, UCL Queen Square Institute of Neurology, London, & Chalfont Centre for Epilepsy, Chalfont St Peter, United Kingdom, **16** Stichting Epilepsie Instellingen Nederland, Heemstede, Netherlands, **17** Department of Neurology, West China Hospital, Sichuan University, Chengdu, China, **18** Department of Psychiatry, University of Oxford, Oxford, United Kingdom, **19** The Alan Turing Institute, London, United Kingdom.
