# Supplementary figures and images for: Evaluating the generalisability of region-naïve machine learning algorithms for the identification of epilepsy in low-resource settings
Source: PLOS Digit Health. 2025 Feb 12;4(2):e0000491. doi: 10.1371/journal.pdig.0000491 (PMC11819582; doi:10.1371/journal.pdig.0000491)

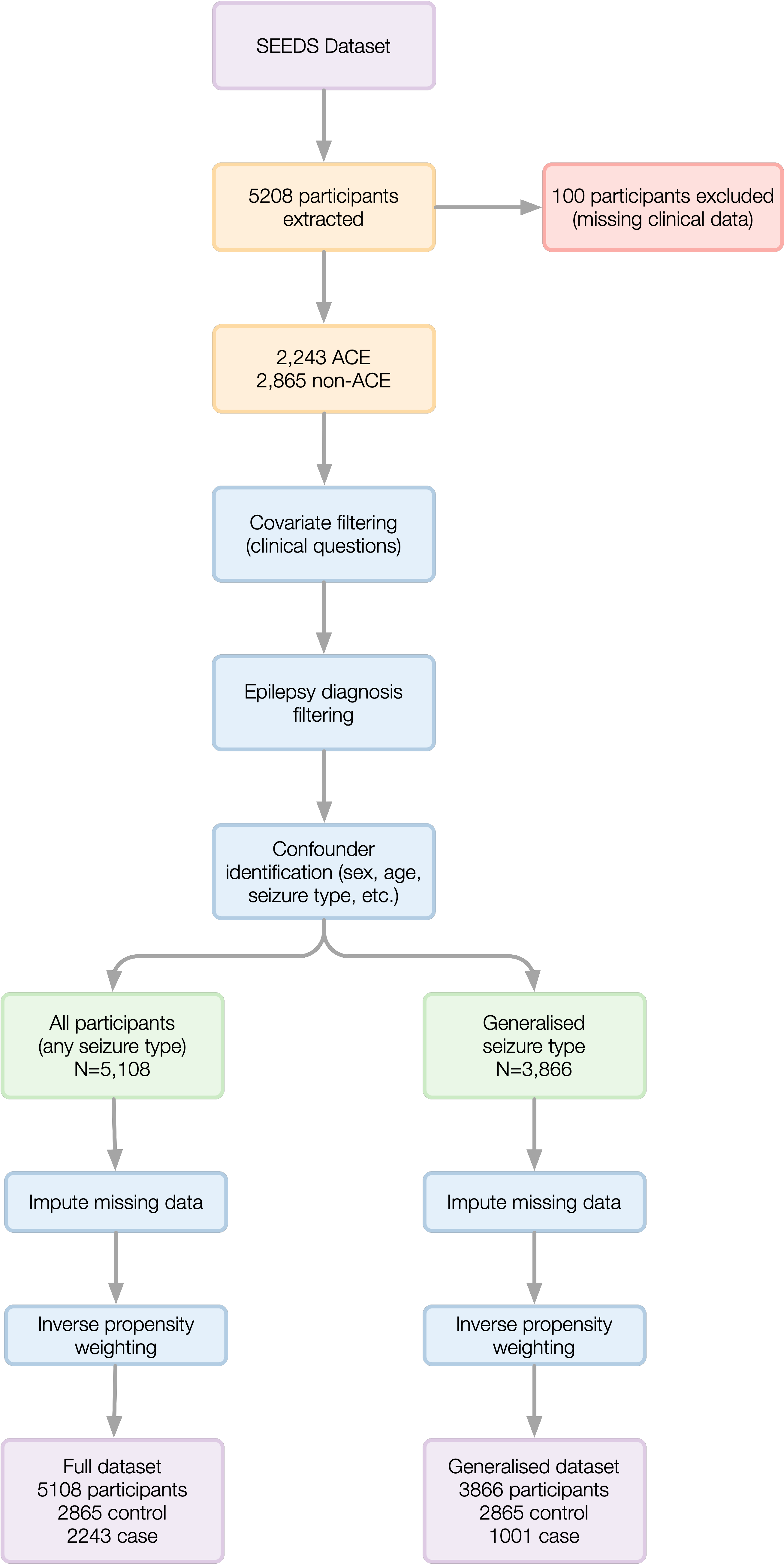

Supplement: S1 Fig — Flowchart showing the process of data preparation (see Table 1). (TIFF) [file pdig.0000491.s001.tiff]

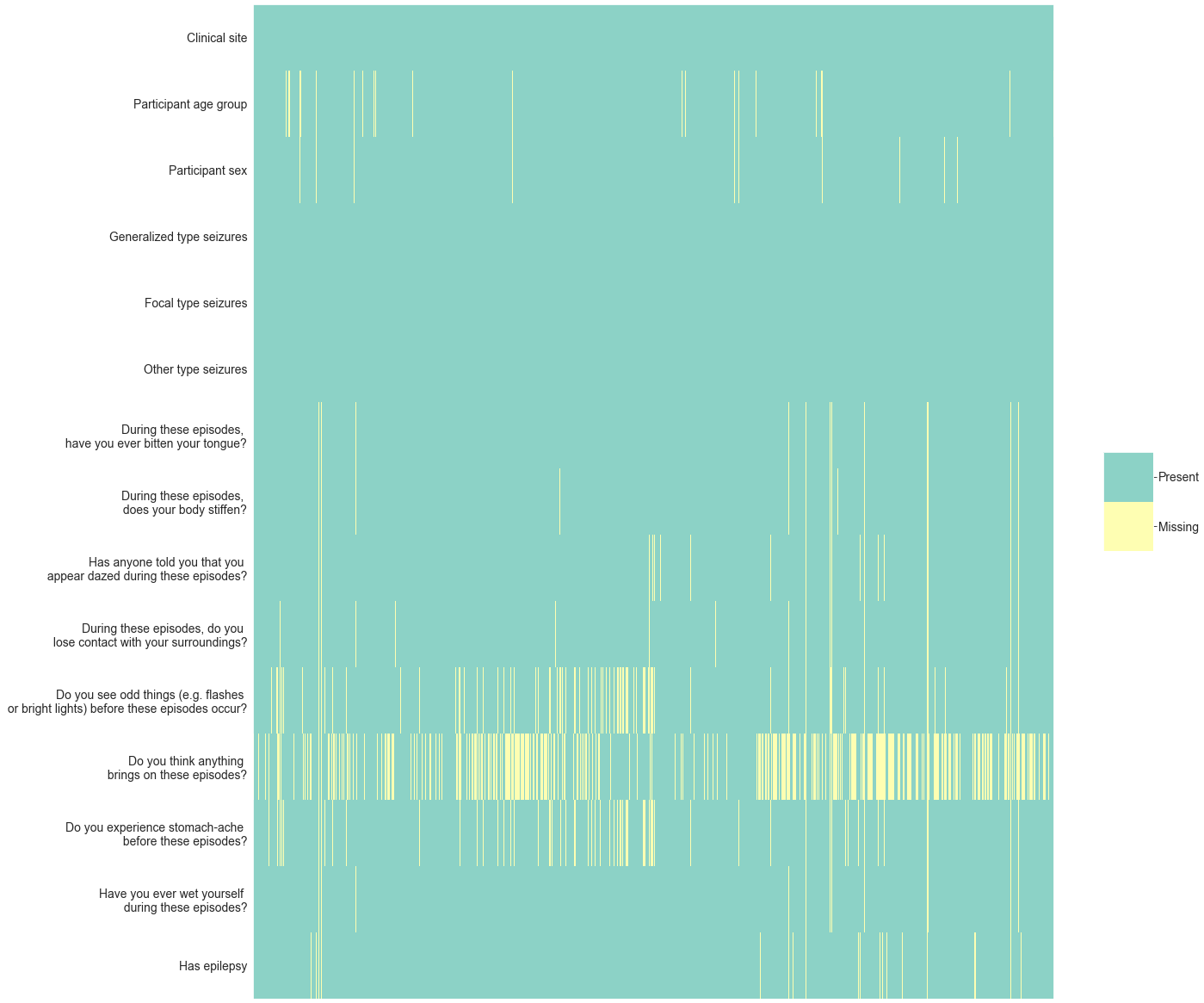

Supplement: S2 Fig — Heatmap showing where there was unexplained missingness in the data before cleaning. Missing values are shown in yellow, others in green. The data are sorted according to assessment date, in ascending order from earliest to latest. (TIFF) [file pdig.0000491.s002.tiff]

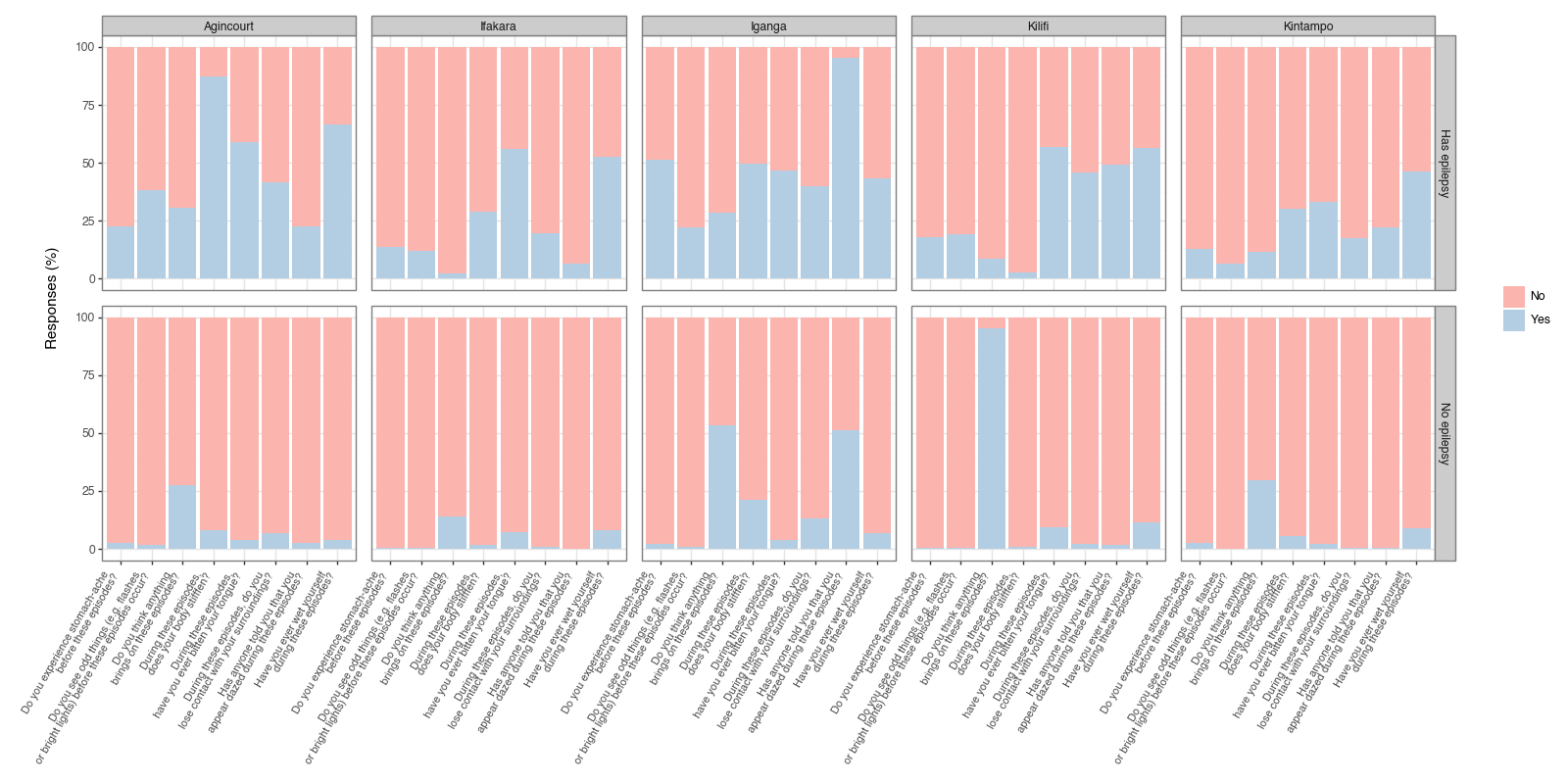

Supplement: S3 Fig — Stacked bar chart showing the percentage of yes/no answers to the covariate questions. Values taken from the cleaned data, split by site and epilepsy diagnostic class. (TIFF) [file pdig.0000491.s003.tiff]

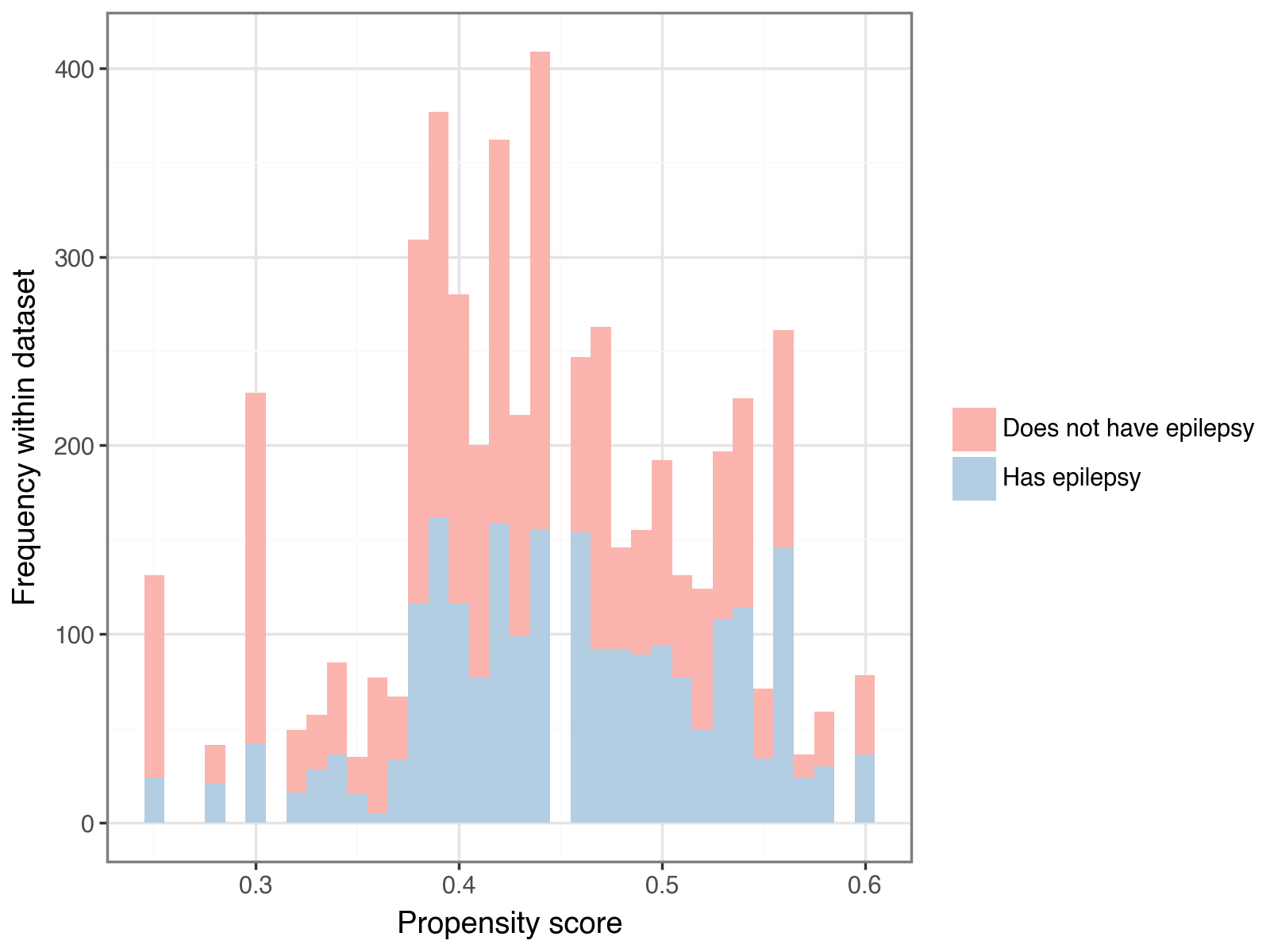

Supplement: S4 Fig — There is a complete overlap–the range of scores for the two diagnostic classes overlaps completely. (TIFF) [file pdig.0000491.s004.tiff]
