# Supplementary material for: Evaluating the generalisability of region-naïve machine learning algorithms for the identification of epilepsy in low-resource settings
Source: PLOS Digit Health. 2025 Feb 12;4(2):e0000491. doi: 10.1371/journal.pdig.0000491 (PMC11819582; doi:10.1371/journal.pdig.0000491)
Supplement: S1 Table — Table summarizing the p-values of the statistical tests of the performances resulting from the testing of each one-site model on each of the other sites in turn. The two samples in each test were the internal performances for that site (from 5-fold cross validation) and the performances of that model on each other site. This was done for each possible combination of the three model types (Logistic Regression, SVM using a linear kernel, and Naive Bayes assuming a Bernoulli distribution) and the two datasets (the whole dataset, and that with the positive cases limited to participants with generalised epilepsy). Thus, there are 6 values for each site. The 2-sample Kolmogorov-Smirnov test has the null hypothesis that the two samples are drawn from the same distribution. Statistical insignificance is taken as insufficient evidence to reject this. There were 7 significant tests for the whole dataset and 4 for the generalised seizure dataset. Agincourt and Kilifi each had 5 significant tests, while the other 3 sites each had at least 5 insignificant tests. (DOCX) [file pdig.0000491.s005.docx]

| Site | Dataset | Significance at 0.05 threshold | Number of such p-values |
| --- | --- | --- | --- |
| Agincourt | All | Significant | 3 |
|  | Generalized | Insignificant | 1 |
|  |  | Significant | 2 |
| Ifakara | All | Insignificant | 3 |
|  | Generalized | Insignificant | 3 |
| Iganga | All | Insignificant | 2 |
|  |  | Significant | 1 |
|  | Generalized | Insignificant | 3 |
| Kilifi | All | Significant | 3 |
|  | Generalized | Insignificant | 1 |
|  |  | Significant | 2 |
| Kintampo | All | Insignificant | 3 |
|  | Generalized | Insignificant | 3 |
| Supplementary Table 1. Kolmogorov-Smirnov test p-values. Table summarizing the p-values of the statistical tests of the performances resulting from the testing of each one-site model on each of the other sites in turn. The two samples in each test were the internal performances for that site (from 5-fold cross validation) and the performances of that model on each other site. This was done for each possible combination of the three model types (Logistic Regression, SVM using a linear kernel, and Naive Bayes assuming a Bernoulli distribution) and the two datasets (the whole dataset, and that with the positive cases limited to participants with generalised epilepsy). Thus, there are 6 values for each site. The 2-sample Kolmogorov-Smirnov test has the null hypothesis that the two samples are drawn from the same distribution. Statistical insignificance is taken as insufficient evidence to reject this. There were 7 significant tests for the whole dataset and 4 for the generalised seizure dataset. Agincourt and Kilifi each had 5 significant tests, while the other 3 sites each had at least 5 insignificant tests. | | | |
