# Supplementary material for: Evaluating the generalisability of region-naïve machine learning algorithms for the identification of epilepsy in low-resource settings
Source: PLOS Digit Health. 2025 Feb 12;4(2):e0000491. doi: 10.1371/journal.pdig.0000491 (PMC11819582; doi:10.1371/journal.pdig.0000491)
Supplement: S2 Table — (DOCX) [file pdig.0000491.s006.docx]

| Parameter | Description |
| --- | --- |
| Sociodemographic variables | Age, sex, education, employment status, marital status |
| Perinatal events | Difficulties feeding, crying, or breathing after birth; abnormal antenatal period |
| Historical risk factors | Head injuries, hospital admissions (e.g., for malaria), diet (e.g., cassava consumption) |
| Clinical history | Seizure frequency, type, and duration |
| Parasitic exposure | Presence of antibodies for Toxocara canis, Toxoplasma gondii, Onchocerca volvulus, etc. |
| Nutritional status | Malnutrition indicators (e.g., body mass index, Z scores) |

Supplementary Table 2. Summary table of variables present in the data.
