# Supplementary material for: Evaluating the generalisability of region-naïve machine learning algorithms for the identification of epilepsy in low-resource settings
Source: PLOS Digit Health. 2025 Feb 12;4(2):e0000491. doi: 10.1371/journal.pdig.0000491 (PMC11819582; doi:10.1371/journal.pdig.0000491)
Supplement: S3 Table — (DOCX) [file pdig.0000491.s007.docx]

|  | Study site | | | | | | |
| --- | --- | --- | --- | --- | --- | --- | --- |
| Convulsive epilepsy predictor | Agincourt | Ifakara | Iganga | Kilifi | Kintampo | Median | p-value |
| Do you experience stomach ache before these episodes? | 0.7 | 1.6 | 2.1 | 2.3 | 1.1 | 1.6 | 0.001 |
| Do you see odd things (e.g. flashes or bright lights) before these episodes occur? | 1.4 | 1.9 | 1.3 | 1 | 0.8 | 1.3 | <0.001 |
| Do you think anything brings on these episodes? | -0.5 | -4.3 | -2.8 | -4.3 | -2 | -2.8 | <0.001 |
| During these episodes, do you lose contact with your surroundings? | 1.5 | 2.3 | 0.4 | 1.2 | 2.6 | 1.5 | <0.001 |
| During these episodes, does your body stiffen? | 2.8 | 2.8 | 0.4 | -0.1 | 1.7 | 1.7 | <0.001 |
| During these episodes, have you ever bitten your tongue? | 0.9 | 2.5 | 0.9 | 0.8 | 2.5 | 0.9 | <0.001 |
| Has anyone told you that you appear dazed during these episodes? | 0.8 | 1 | 3.2 | 1.3 | 3 | 1.3 | <0.001 |
| Have you ever wet yourself during these episodes? | 1.5 | 1.9 | 1.6 | 1.3 | 2.2 | 1.6 | <0.001 |
| Supplementary Table 3. Mean predictor weight values and associated p-values | | | | | | | |
